# Shock attenuation in shoes compared to barefoot: a systematic review

**DOI:** 10.1186/1757-1146-5-S1-O1

**Published:** 2012-04-10

**Authors:** Alycia Fong Yan, Claire Hiller, Peter Sinclair, Richard Smith

**Affiliations:** 1 Faculty of Health Science, The University of Sydney, Sydney, NSW, 2041, Australia; 2 Discipline of Physiotherapy, The University of Sydney, Sydney, NSW, 2041, Australia

## Background

The debate over the advantages and disadvantages of barefoot versus shod running has gained momentum recently [1,2] with the retail market aiming to mimic the motion of the foot during barefoot gait[3]. The aim of this study was to conduct a systematic review of articles that compared shock attenuation in the shod condition to barefoot during weight bearing activity in healthy individuals.

## Materials and methods

The major databases were searched for the following keywords: *barefoot*, *foot*, *feet*, boot*, shoe*, *impact*, *shock*, *pressure*, *force*, *viscoelastic*, and *insert.* Articles were screened with inclusion and exclusion criteria set *a priori*. Articles were grouped according to shoe type and where possible, a meta-analysis was used.

## Results

Thirty-eight articles were found with 27 articles examining athletic shoes compared to barefoot. For running, footwear attenuated loading rate and tibial acceleration (Table 1). In contrast, the use of shoes increased vertical ground reaction forces (vGRF) during running (Table 1) and walking when measured at the impact transient. Results varied significantly in favour of the shod or barefoot condition depending on whether data was collected at the impact transient or the peak. Thirteen articles did not report the footfall technique, while two studies reported variable technique.

**Table 1:** Pooled effect of bare feet vs. athletic footwear during running (+’ve: attenuated in BF, –‘ve: attenuated in shod)

Variable	Time of Collection	# of Studies	*n*	Mean Difference [95% CI]	P Value
Vertical Ground Reaction Force	Impact Transient	De Wit et al 2000, Divert et al 2005, Esnault 1985, Lieberman et al 2010	108	0.22 [0.20, 0.23]	<0.00001
	
	Peak Force	Alcantara et al 1996, Braunstein et al 2010, Dickinson et al 1986, Fong et al 2007, Kerrigan et al 2009, Serrao & Amadio 2001, Squadrone & Gallozzi 2009,Stockton & Dyson 1998	128	-0.03 [-0.07, 0.01]	0.19

Loading Rate	Impact Transient	De Wit 2000, Lieberman 2010	72	-3.56 [-4.10, -3.02]*	<0.00001

	Peak Force	Alcantara 1996, Serrao & Amadio 2001	11	-0.59 [-2.52, 1.35]*	0.55

Tibial Acceleration	Peak Force	Alcantara et al 1996, McNair & Marshall 1994	18	-3.19 [-4.35, -2.03]*	<0.00001

*Standardised Mean Difference [95% CI]

## Conclusions

Evidence suggests the shock absorbing properties of athletic footwear are effective during jump landings. Results varied significantly in favour of the shod or barefoot condition depending on whether data was collected at the impact transient or the peak. Footfall technique appears to have a significant effect on vertical ground reaction force. Activity-specific designs for footwear should take into account the region of the shoe which absorbs the initial impact. Attention should be given to develop consistent protocols for examining shock attenuation in footwear research.
